# Response to the Letter to Editor

**Published:** 2018

**Authors:** Yuji Tsutsui

**Affiliations:** BSc, Division of Radiology, Department of Medical Technology, Kyushu University Hospital, Fukuoka, Japan

We read with great interest the letter to the editor and the article by Nakahara et al. (Nakahara T, Daisaki H, Yamamoto Y, Iimori T, Miyagawa K, Okamoto T, et al. Use of a digital phantom developed by QIBA for harmonizing SUVs obtained from the state-of-the-art SPECT/CT systems: a multicenter study. EJNMMI Research. 2017;7:53.) (1). It is embarrassing that we did not recognize their paper in an academic journal of EJNMMI Research. I was ashamed of our ignorance with their works, although their focus in SPECT was different with ours with PET.

The digital phantom has been widely used in nuclear medicine technology field for a long time (1-3). We also used it in our previous study (4). Quantitative Imaging Biomarkers Alliance (QIBA), an organization to improve the quantitative accuracy of imaging biomarkers, generated a digital phantom as a standard for a PET oncology (5). This phantom is introduced on the website, and is open and freely available to be used for examinations. On the other hand, root mean squared error (RMSE) is a frequently used measure of the differences between values observed. RMSE is a simple mathematical measure and has been used for long time in nuclear medicine and molecular imaging technique with an identical formula (6-8). Based on these backgrounds, it is no wonder that many researchers may conceive to investigate the quantitative accuracy of images with a combination of RMSE and QIBA phantom.

Although we did not recognize the article by Nakahara et al, we may be better to cite their article in our manuscript.

Thank you for your understanding.

## Conflicts of interest

None declared.
